# Quantification of the Cannabinoid Type 1 Receptor Availability in the Mouse Brain

**DOI:** 10.3389/fnana.2020.593793

**Published:** 2020-11-20

**Authors:** Isabelle Miederer, Viktoria Wiegand, Nicole Bausbacher, Petra Leukel, Stephan Maus, Manuela A. Hoffmann, Beat Lutz, Mathias Schreckenberger

**Affiliations:** ^1^Department of Nuclear Medicine, University Medical Center of the Johannes Gutenberg University Mainz, Mainz, Germany; ^2^Institute of Neuropathology, University Medical Center of the Johannes Gutenberg University Mainz, Mainz, Germany; ^3^Department of Occupational Health and Safety, Federal Ministry of Defense, Bonn, Germany; ^4^Institute of Physiological Chemistry, University Medical Center of the Johannes Gutenberg University Mainz, Mainz, Germany; ^5^Leibniz Institute for Resilience Research, Mainz, Germany

**Keywords:** cannabinoid type 1 receptor, [^18^F]MK-9470, microPET, mouse, immunohistochemistry

## Abstract

**Introduction**: The endocannabinoid system is involved in several diseases such as addictive disorders, schizophrenia, post-traumatic stress disorder, and eating disorders. As often mice are used as the preferred animal model in translational research, in particular when using genetically modified mice, this study aimed to provide a systematic analysis of *in vivo* cannabinoid type 1 (CB1) receptor ligand-binding capacity using positron emission tomography (PET) using the ligand [^18^F]MK-9470. We then compared the PET results with literature data from immunohistochemistry (IHC) to review the consistency between *ex vivo* protein expression and *in vivo* ligand binding.

**Methods**: Six male C57BL/6J (6–9 weeks) mice were examined with the CB1 receptor ligand [^18^F]MK-9470 and small animal PET. Different brain regions were evaluated using the parameter %ID/ml. The PET results of the [^18^F]MK-9470 accumulation in the mouse brain were compared with immunohistochemical literature data.

**Results**: The ligand [^18^F]MK-9470 was taken up into the mouse brain within 5 min after injection and exhibited slow kinetics. It accumulated highly in most parts of the brain. PET and IHC classifications were consistent for most parts of the telencephalon, while brain regions of the diencephalon, mesencephalon, and rhombencephalon were rated higher with PET than IHC.

**Conclusions**: This preclinical [^18^F]MK-9470 study demonstrated the radioligand’s applicability for imaging the region-specific CB1 receptor availability in the healthy adult mouse brain and thus offers the potential to study CB1 receptor availability in pathological conditions.

## Introduction

The endocannabinoid system plays an important role in several physiologic processes such as memory function, motor control, pain processing, food intake, and energy balance. It is composed of cannabinoid type 1 (CB1) and 2 receptors, its endogenous ligands (endocannabinoids), and their synthesizing and degrading enzymes. The CB1 receptor is a G-protein coupled receptor and is prominently located presynaptically on excitatory and inhibitory neurons. In the brain of rodents, rhesus monkeys as well as humans, CB1 receptor protein is found at very high density in regions such as the globus pallidus, substantia nigra, hippocampal dentate gyrus, and the cerebellar cortex. A high density of CB1 receptors is also observed in the cerebral cortex, other parts of the hippocampal formation, and striatum as shown by autoradiographic studies (Herkenham et al., 1990). A sparse to a very low density of receptors was observed in regions such as the hypothalamus, basal amygdala, central gray, thalamus, and brainstem (Herkenham et al., 1990). In a wide range of preclinical and clinical positron emission tomography (PET) studies, altered availability of CB1 receptor has been shown in the context of psychiatric diseases, such as addictive disorders (Gérard et al., 2010; Hirvonen et al., 2012, 2013, 2018; Neumeister et al., 2012; Ceccarini et al., 2013b, 2014, 2015; D’Souza et al., 2016), schizophrenia (Wong et al., 2010; Ceccarini et al., 2013a; Verdurand et al., 2014; Ranganathan et al., 2016), post-traumatic stress disorder (Neumeister et al., 2013; Pietrzak et al., 2014) and eating disorders (Addy et al., 2008; Gérard et al., 2011; Casteels et al., 2014; Ly et al., 2015; Ceccarini et al., 2016; Lahesmaa et al., 2018), furthermore in neurological diseases, such as Parkinson’s disease (Casteels et al., 2010b,d; Van Laere et al., 2012; Ceccarini et al., 2019b), Huntington’s disease (Casteels et al., 2010c, 2011; Ooms et al., 2014; Ceccarini et al., 2019a) and epilepsy (Goffin et al., 2008, 2011; Cleeren et al., 2018). A better understanding of the endocannabinoid system with its receptors will help to refine diagnostic and evidence-based therapeutic strategies for the treatment of associated disorders.

For imaging, the endocannabinoid system with PET, several ^11^C- and ^18^F-labeled compounds have been developed. Established CB1 receptor ligands include [^18^F]MK-9470 (Liu et al., 2007), [^18^F]FMPEP-d2 (Donohue et al., 2008), [^11^C]MePPEP (Donohue et al., 2008), [^11^C]SD5024 (Tsujikawa et al., 2014), and [^11^C]OMAR ([^11^C]JHU75528; Fan et al., 2006). In the absence of an on-site cyclotron, we choose the ^18^F-labeled ligand MK-9470 for our studies. [^18^F]MK-9470 was developed by Merck and Company Incorporation based on the chemical structure of taranabant (Merck and Company Incorporation) and has a high affinity to the CB1 receptor (IC_50_ = 0.7 nM), high lipophilicity (logD_7.*3*_ = 4.7), and a good brain uptake. The signal-to-noise ratio in PET images of rhesus monkeys and humans is 4–5:1 (Burns et al., 2007; Liu et al., 2007). Recent studies showed that [^18^F]MK-9470 proved to be well suited for imaging CB1 receptor availability in rats, monkeys as well as humans in healthy and pathological conditions.

To gain a better understanding of the operation of the endocannabinoid system, many preclinical studies have been conducted. Using the ligand [^18^F]MK-9470, almost all preclinical studies were carried out in rats; for certain experimental questions, however, only mouse models are suitable, for example when using genetically modified mice. Therefore, this study aimed to provide a systematic analysis of regions in the mouse brain using PET using the ligand [^18^F]MK-9470. Also, we compared our results with literature data from immunohistochemistry (IHC) to review the consistency between *ex vivo* and *in vivo* methods.

## Materials and Methods

### Animals

Six male C57BL/6J mice (21.5–28.1 g, 6–9 weeks of age; obtained from the Translational Animal Research Center—TARC of the University Medical Center Mainz) were examined. All applicable international, national, and/or institutional guidelines for the care and use of animals were followed. This study was approved by the respective state authorities (Landesuntersuchungsamt Rheinland-Pfalz).

### Radiolabeling of [^18^F]MK-9470

The synthesis of the precursor [N-[(1S, 2S)-2-(3-Cyanophenyl)-3-(4-hydroxyphenyl)-1-methylpropyl]-2-methyl-2-[(5-methylpyridin-2-yl)oxy]propanamide was conducted as described in detail by Liu et al. (2007). For radiolabeling, the phenol group of the precursor was deprotonated with cesium carbonate in dimethylformamide and finally reacted with [^18^F]fluoroethyl tosylate (PET Net GmbH, Erlangen, Germany) in a nucleophilic reaction as previously described by our group (Miederer et al., 2013). Usually, the synthesis took 30 min, including the time for reversed-phase HPLC for purification and subsequent separation from the organic solvent by C18 cartridge purification.

### PET Data Acquisition

Isoflurane inhalation anesthesia (2% isoflurane vaporized in 60% oxygen) was used to immobilize the mice, which were positioned in the small animal PET scanner in the headfirst prone position. A venous catheter, which was placed in one of the tail veins, was used to inject [^18^F]MK-9470. Together with the injection, a 60-min PET measurement was started with a Focus 120 small animal PET scanner (Siemens/CTI, Knoxville, TN, USA). This small animal PET scanner has lutetium oxyorthosilicate detectors having a size of 1.5 × 1.5 × 10 mm^3^ for coincidence detection of photons (time window: 6 ns). The resolution in the center of the field of view is ≤1.4 mm. As part of the quality control, a detector normalization and cross-calibration with a dose calibrator (VDC 404, Veenstra Instruments, Joure, Netherlands) were performed regularly. The PET data acquisition took place in the list mode data format.

### PET Data Analyses

Two mice, a CB1 receptor-deficient mouse, and a wild-type mouse, whose data acquisition is described in Miederer et al. (2013), were re-analyzed. This means that these data, which were previously only analyzed statistically, are presented over the whole acquisition time course.

The list-mode data, acquired for this study, were sorted into a sinogram matrix with frames of 3 × 20, 3 × 60, 3 × 120, 10 × 300 s (=19 frames). The data were reconstructed with filtered back-projection [ramp filter (cut-off = 0.5)] into a 128 × 128 matrix with 95 slices of 0.8 mm thickness (pixel size 0.87 × 0.87 mm^2^). Corrections included detector dead time and random coincidences, which yielded images in the unit Bq/ml.

PET data were co-registered to the T2-weighted magnetic resonance image (MRI) template provided by the PMOD software (version 4.0, Zurich, Switzerland) based on the work of Ma et al. (2005) and Mirrione et al. (2007). The following volumes-of-interest (VOI) were selected from the VOI atlas (Ma et al., 2005 and Mirrione et al., 2007): (1) forebrain: olfactory bulb, caudate putamen (striatum), basal telencephalon septum, cerebral cortex, hippocampus, thalamus, hypothalamus, amygdala; (2) midbrain: superior colliculus, inferior colliculus, central substantia grisea, midbrain; (3) hindbrain: cerebellum, brain stem; and (4) whole brain (created from the previously defined 15 brain regions). The outcome parameter was calculated as %ID/ml = mean value of the radioactivity concentration (in the unit Bq/ml)/injected radioactivity (in the unit Bq) × 100% and included the 40–60 min acquisition interval for individual brain regions. All results were reported as mean ± standard deviation. A two-tailed paired *t*-test was used to compare means for VOI of different receptor densities and thus, four main comparisons were defined before the analyses: cerebellum vs. pons, caudate putamen vs. pons, cerebellum vs. hippocampus, and hippocampus vs. pons. The global significance level was α_global_ = 0.05 and a Bonferroni correction yielded a local significance level of α_local_ = 0.0125.

### Classification of PET Data and IHC Literature Data

The PET results of the [^18^F]MK-9470 accumulation in the mouse brain were compared with the immunohistochemical work of Egertová et al. (2003), Harkany et al. (2003), and Cristino et al. (2006). The methods used in each case are briefly explained below. Egertová et al. (2003) assessed the role of the enzyme fatty acid amide hydrolase (FAAH) in the regulation of endocannabinoid signaling, thereby comparing the distribution of FAAH and CB1 receptor expression in the brains of 129SvJ-C57BL/6 mice. In brief, Egertová et al. (2003) used brain sections that were preincubated with normal goat serum. To depict the CB1 receptor immunoreactivity, one part of the brain sections was incubated with the antiserum 2825.3 to the C-terminal tail of the mouse/rat CB1 receptor and the other with affinity-purified antibodies from the CB1 receptor-antiserum 2816.4. Harkany et al. (2003) analyzed the distribution of CB1 receptors, vesicular glutamate transporters 3, and FAAH in the basal forebrain of C57BL/6N mice. In short, to depict the CB1 receptor immunoreactivity in a double-labeling experiment, Harkany et al. (2003) used brain sections that were preincubated with normal donkey serum and then incubated with rabbit anti-CB1 receptor primary antibodies raised against the C-terminal tail of the CB1 receptor. Subsequently, the brain sections were incubated with carbocyanine (Cy)2-conjugated donkey anti-rabbit immunoglobulin G. To depict the CB1 receptor immunoreactivity in a triple-labeling experiment, brain sections were preincubated with normal donkey serum and then incubated with rabbit and goat anti-CB1 receptor primary antibodies directed against the C-terminal tail of the rat CB1 receptor. Subsequently, the brain sections were incubated with carbocyanine (Cy)2-, 3- and 4-conjugated antibodies from donkeys. Cristino et al. (2006) investigated the localization of CB1 and transient receptor potential vanilloid type 1 channel (TRPV1) in Swiss and ABH (wild-type, CB1^−/−^, and TRPV1^−/−^) mice. In brief, Cristino et al. (2006) used brain sections that were preincubated with normal goat serum and then incubated with rabbit polyclonal antibodies raised against the N-terminal tail of the CB1 receptor. Subsequently, the brain sections were incubated in biotinylated goat anti-rabbit immunoglobulin G.

To facilitate the comparison between (quantitative, continuous) PET data and (qualitative) IHC data, the data were classified into four categories, namely no (−), low (+), mean (++) and high (+++) accumulation of the radioactivity concentration or immunohistochemical staining. The IHC data were classified based on visual inspections of the results and their descriptions in the literature sources. Statements such as “little or no,” “few” or “void” led to a classification of “–.” Statements like “low,” “some” or “weak” were classified as “+.” Descriptions like “network of CB1-immunoreactive fibers” or “intensely CB1 receptor-ir fiber meshwork” led to a rating of “++” and statements like “dense meshwork of fibers” or “very high concentration of CB1-immunoreactivity” were rated as “+++.” The PET data were classified based on the outcome parameter %ID/ml, ranging from mean values of 1.5–2.6 %ID/ml, and were subdivided into three categories. That is, signals within the interval 1.5–1.8 %ID/ml lead to a rating of “+.” Parameters within the interval 1.8–2.2 %ID/ml were rated as “++” and signals within the interval 2.2–2.6 %ID/ml were classified as “+++.” As there was no brain region devoid of a PET signal, the category “−” was omitted here.

## Results

**Figure 1:**
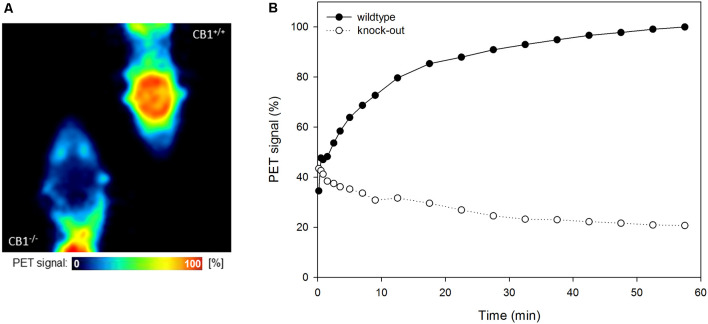
[^18^F]MK-9470 binds specifically to cannabinoid type 1 (CB1) receptors in the mouse brain. **(A)** Left: CB1 receptor knock-out mouse, right: wild-type mouse. Positron emission tomography (PET) images were summed from 40 to 60 min [injected radioactivity: 8.5 MBq (CB1^−/−^) and 8.0 MBq (CB1^+/+^), anaesthesia: 0.2 ml xylazine/ketamine, data acquisition: 60 min]. **(B)** Standardized volumes-of-interest (VOI) were drawn for the whole mouse brains (*n* = 2). Extracted radioactivity concentrations were normalized to the maximum radioactivity concentration in the wild-type mouse.

[^18^F]MK-9470 binds specifically to CB1 receptors in the mouse brain, as depicted in Figures 1A,B. In the wild-type mouse, the ligand is slowly taken up into the brain, while in the knock-out mouse, it is washed out after the first half minute. The level of the unspecific signal in the CB1 receptor knock-out mouse is about 20% of the maximum signal measured in the wild-type mice.

**Figure 2:**
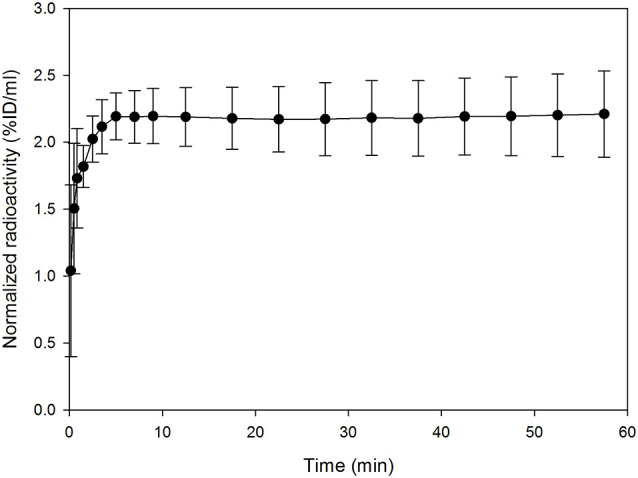
The ligand [^18^F]MK-9470 exhibits slow kinetics in the mouse brain. Standardized VOI were drawn for the whole mouse brains (PMOD Technologies LLC, Zurich, Switzerland). Extracted radioactivity concentrations were normalized to the injected radioactivity for each mouse and averaged over the group (*n* = 5; injected radioactivity: 6.6 ± 1.1 MBq, anesthesia: 2% isoflurane). Results are presented as mean ± standard deviation.

The radioactivity concentration of [^18^F]MK-9470 was taken up into the mouse brain within 5 min after injection, as illustrated in Figure 2. Then, it reached its maximum and remained at a mean value of approximately 2.2 %ID/ml until the end of the acquisition; the coefficient of variation is approximately 12% in this time interval. As expected, the ligand [^18^F]MK-9470 exhibited slow kinetics in the mouse brain.

**Figure 3:**
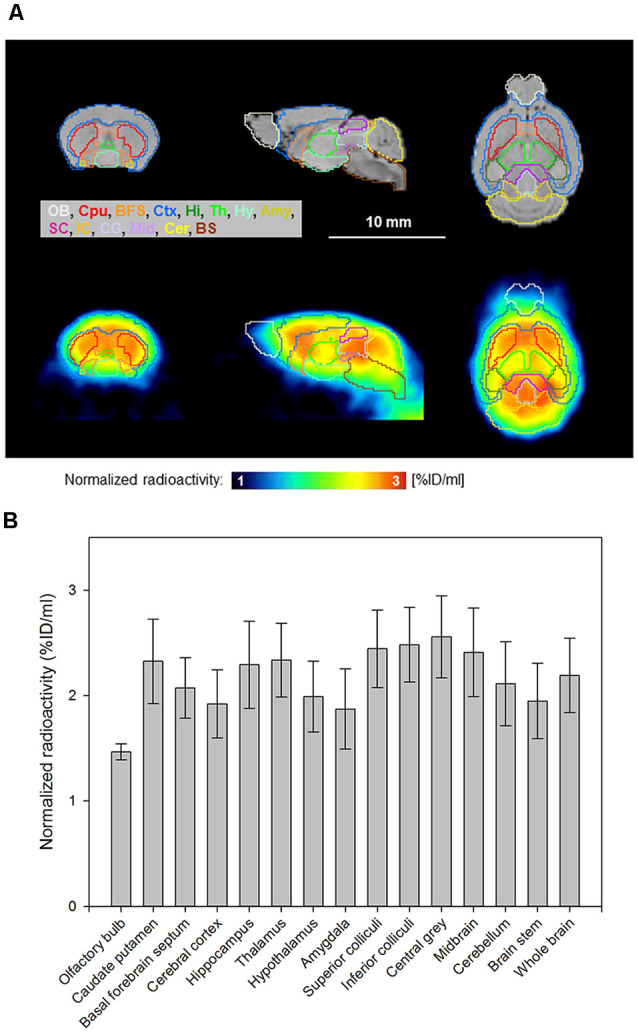
[^18^F]MK-9470 accumulation in the mouse brain as measured with PET. PET images were summed from 40 to 60 min and co-registered to a T2-weighted magnetic resonance template (PMOD Technologies LLC, Zurich, Switzerland). The radioactivity concentration of the ligand in tissue was normalized to the injected radioactivity for each mouse and averaged over the entire group (*n* = 5; injected radioactivity: 6.6 ± 1.1 MBq, anesthesia: 2% isoflurane, data acquisition: 60 min). **(A)** Horizontal brain layers of magnetic resonance images (MRI) and PET images are passing through the interaural line from 6 to 0 mm. T2-weighted magnetic resonance template images are presented as anatomic reference. **(B)** Region-specific accumulation of [^18^F]MK-9470 in the mouse brain as obtained from standardized VOIs (PMOD Technologies LLC, Zurich, Switzerland). Results are presented as mean ± standard deviation.

Reconstructed PET images showed the accumulation of [^18^F]MK-9470 in the mouse brain and differences between brain regions, as depicted in Figures 3A,B. Calculated from summed images (40–60 min), the highest signal was obtained in the central gray (2.56 ± 0.39 %ID/ml) and the lowest in the olfactory bulb (1.47 ± 0.08 %ID/ml). The coefficients of variation for all brain regions were in the range of 5–20%. The descending rank order of [^18^F]MK-9470 concentration in defined brain regions was: central gray > inferior colliculi > superior colliculi > midbrain > thalamus > caudate-putamen > hippocampus > whole-brain > cerebellum > basal forebrain septum > hypothalamus > brain stem > cortex cerebri > amygdala > olfactory bulb. The two-sided paired *t*-tests revealed significant differences, that is, *p*-values < 0.0125, for cerebellum vs. brain stem, caudate putamen vs. brain stem, cerebellum vs. hippocampus, and hippocampus vs. brain stem.

The comparison of PET and IHC data is given in Table 1. Classifications were consistent for the brain region’s olfactory bulb, cerebral cortex, hippocampus, basal forebrain septum, and amygdala. The other brain regions showed a higher rating for PET data as compared to IHC data. PET and IHC classifications matched for most parts of the telencephalon, whereas brain regions of the diencephalon, mesencephalon, and rhombencephalon were rated higher with PET.

**Table 1:** Comparison of positron emission tomography and immunohistochemistry data.

	[^18^F]MK-9470 %ID/ml	Category of [^18^F]MK-9470 accumulation^1^	Category of immune reaction^2^
Telencephalon			
Olfactory bulb	1.47 ± 0.08	+	+
Cerebral cortex	1.92 ± 0.32	++	++
Hippocampus	2.29 ± 0.41	+++	+++
Basal ganglia: caudate putamen	2.33 ± 0.40	+++	+
Basal ganglia: globus pallidus	n/a	n/a	+++
Basal forebrain septum	2.07 ± 0.29	++	+/++
Amygdala	1.87 ± 0.38	++	++
Diencephalon			
Hypothalamus	1.99 ± 0.34	++	+
Thalamus	2.34 ± 0.35	+++	−
Mesencephalon			
Tectum mesencephalic:colliculus superior	2.44 ± 0.37	+++	−
Tectum mesencephalic: colliculus inferior	2.48 ± 0.35	+++	−
Tegmentum mesencephalic:substantia nigra	n/a	n/a	+++
Substantia grisea centralis	2.56 ± 0.39	+++	+
Mesencephalon (whole region)	2.41 ± 0.42	+++	+
Rhombencephalon			
Cerebellar cortex	n/a	n/a	+++
Cerebellum (whole region)	2.11 ± 0.40	++	n/a
Brain stem	1.95 ± 0.36	++	−
Whole-brain	2.19 ± 0.35	++	n/a

*No (–), low (+), mean (++) and high (+++) accumulation of the radioactivity concentration or immunohistochemical staining. ^1^Classification of the cannabinoid type 1 (CB1) receptor accumulation is based on the range of the outcome parameter %ID/ml. ^2^Classification of the CB1 receptor density into categories is based on the results of Egertová et al. (2003), Harkany et al. (2003), and Cristino et al. (2006), n/a: no data available*.

## Discussion

We present a PET study of the CB1 receptor *in vivo* availability in the mouse brain and compare the results with literature data from IHC. We understand this work as a methodological basis for further studies in mouse disease models.

The ligand [^18^F]MK-9470 shows a high affinity to the CB1 receptor, high lipophilicity, and a good uptake in the brain. It is based on the chemical structure of the pharmaceutical taranabant (MK-0364; Merck and Company Incorporation), which is an inverse agonist just like the earlier developed pharmaceutical rimonabant (SR141716; Sanofi-Aventis). Both ligands had the indication for the treatment of obesity and entered phase III clinical trials; however, they had to be withdrawn from clinical trials due to serious adverse events. Both ligands are nevertheless candidates in preclinical PET studies for investigation of the endocannabinoid system (Hjorth et al., 2016).

In a comparative study in CB1 receptor knock-out and wild-type mice, we showed that [^18^F]MK-9470 specifically binds to CB1 receptors in the evaluation of the whole brain (Miederer et al., 2013). The analysis of the whole time course of the data showed that unspecific binding is approximately 20% of the signal present in a wild-type mouse during the period of 40–60 min. The specificity for CB1 receptors was also demonstrated in another study in knock-out (*n* = 4) and wild-type mice (*n* = 4) for the ligand [^11^C]OMAR ([^11^C]JHU75528). Here, knock-out mice showed a 50% lower uptake as compared to wild-type mice in this period (Herance et al., 2011). Related to these data, [^18^F]MK-9470 exhibits less unspecific signals.

The ligand [^18^F]MK-9470 was taken up into the mouse brain within 5 min after injection and exhibits slow kinetics, which was observed in rat and human brain studies before. In the rat brain, this ligand arrives at a plateau approximately 300 min after injection (Casteels et al., 2012), whereas in the human brain, this plateau is reached earlier, after approximately 120 min (Burns et al., 2007). It is generally assumed that the slow kinetics of the ligand is caused by the high affinity of the ligand for the receptor and the high density of the receptor in the brain, which might lead to rapid local re-association of the ligand to the receptor. In the context of PET studies, a slow kinetic behavior of a ligand makes it difficult to determine its dissociation rates from the receptors (“k_4_”) using mathematical models, as shown for [^18^F]MK-9470 in previous studies (Sanabria-Bohórquez et al., 2010; Casteels et al., 2012; Miederer et al., 2018). In other mouse studies, ^11^C-labeled CB1 receptor ligands were used, such as [^11^C]MePPEP and [^11^C]OMAR ([^11^C]JHU75528), which could also be classified as slow, but still showed faster kinetics than [^18^F]MK-9470 and could be analyzed with mathematical models (Horti et al., 2006; Terry et al., 2008). The slow kinetics of the ligand [^18^F]MK-9470 would probably not allow the application of mathematical models for mouse brain data; for analyses of the [^18^F]MK-9470 accumulation in the mouse brain with semi-quantitative parameters such as %ID/ml or standardized uptake value (SUV), simple acquisition protocols starting from 10 min after ligand injection are well applicable.

As visually assessed in this study, the ligand [^18^F]MK-9470 accumulated highly in regions of the telencephalon, diencephalon, mesencephalon, and rhombencephalon, and thus showed the same distribution as illustrated in C57BL/6 control animals by Ooms et al. (2014). In our study, we referred to the calculations of the parameter %ID/ml, which partially overlaps in its ranking with that of other ligands (central gray > inferior colliculi > superior colliculi > midbrain > thalamus > caudate-putamen > hippocampus > whole-brain > cerebellum > basal forebrain septum > hypothalamus > brain stem > cortex cerebri > amygdala > olfactory bulb). In a mouse study with [^18^F]FMPEP-d2, in which binding ratios (reference region: thalamus) were calculated as outcome parameter, it was shown that the regions striatum, frontal cortex, and hippocampus were calculated in this order as highly accumulating, while the ranking of other regions, such as hypothalamus, brain, cerebellum, parietotemporal cortex, was age-dependent (Takkinen et al., 2018). In a mouse study with [^11^C]OMAR ([^11^C]JHU75528), the parameter %ID/ml revealed a rank order of striatum > hippocampus > cortex > cerebellum > thalamus > brain stem (Horti et al., 2006). The %ID/ml values were slightly higher, but in the same range (approximately 2.5–6 %ID/ml) as compared to the values for the parameter %ID/ml for the ligand [^18^F]MK-9470 in our study (1.5–2.6 %ID/ml). In a study with [^11^C]MePPEP, however, the authors could not analyze individual brain regions and stated that they measured a similar concentration of the ligand in every brain region (Terry et al., 2008). We assume that these different results are due to the different chemical structures of the ligands, but also due to the comparison of different outcome parameters or age-dependent effects. Interestingly, there is evidence that the level of CB1 receptor protein expression is not necessarily proportional to the efficacy of G protein-dependent signaling of the CB1 receptor. As reviewed by Busquets-Garcia et al. (2018), functional studies indicate that different levels of G protein activations are observed between brain regions and also within the same brain regions. It was shown that the hypothalamus, a region of low levels of the CB1 receptor, induces a stronger G protein activation as compared to brain regions with higher CB1 receptor expression (Breivogel et al., 1997). Furthermore, in the hippocampus of CB1 receptor-deficient mice, glutamatergic neurons were shown to induce a stronger G protein activation as compared to GABAergic interneurons (Steindel et al., 2013). According to Busquets-Garcia et al. (2018), processes related to specific cell types and subcellular compartments could explain the range of behavioral effects induced by exogenous cannabinoids. Returning to the present study in mice, we also asked whether the receptor availability of individual brain regions can be distinguished from each other at all due to the small brain size of mice. Two-sided paired *t*-tests revealed significant differences for various brain regions investigated (cerebellum vs. brain stem, caudate putamen vs. brain stem, cerebellum vs. hippocampus, and hippocampus vs. brain stem), indicating that accumulations of the ligand [^18^F]MK-9470 in the mouse brain can be calculated for individual brain regions.

PET and IHC classifications were consistent for most parts of the telencephalon, while brain regions of the diencephalon, mesencephalon, and rhombencephalon were rated higher with PET than in IHC. This discrepancy applies to the brain regions thalamus, colliculus superior, colliculus inferior, and brainstem, which have no or few receptors, as well as to the brain regions caudate-putamen, substantia grisea centralis, and the entire mesencephalon, whose receptor densities have been described as low or weak. The works of Egertová et al. (2003), Harkany et al. (2003) and Cristino et al. (2006) showed consistent anatomical localization of CB1 receptors. Egertová et al. (2003) observed both complementary and anatomically associated patterns of FAAH and CB1 receptors and concluded implications of FAAH on previously described retrograde signaling of endocannabinoids. Harkany et al. (2003) found similar appearances of CB1 receptors and FAAH as also described by Egertová et al. (2003) and complementary patterns of CB1 receptors and vesicular glutamate transporters three from which they concluded implication on cholinergic signaling mechanisms. The work of Cristino et al. (2006) demonstrates the co-expression of CB1 and TRV1 receptors in several brain regions which explains findings from previous *in vitro* studies. Differences between PET and IHC can be explained, on the one hand, by the lipophilicity of the ligand [^18^F]MK-9470 that leads to levels of unspecific signal in the PET image. In a study with rats using the CB1 receptor antagonist rimonabant for receptor blocking before PET, we estimated that 58% of the signal is allocated to unspecific binding (Miederer et al., 2013); we assume that in this mouse study, too, a significant portion of the signal was due to non-specific binding. One explanation is that the receptor-ligand [^18^F]MK-9470 probably also accumulates in lipophilic cell membranes, which cannot be prevented. On the other hand, isoflurane anesthesia has been shown to affect the accumulation of [^18^F]MK-9470 in the rat brain. Casteels et al. (2010a) showed a reduction of the relative uptake (SUVs normalized to whole brain uptake) in cortical brain regions and an increase of this parameter in subcortical regions, the cerebellum, and pons under isoflurane anesthesia as compared to control animals. The authors showed that pentobarbital produced similar effects. Usually, anesthesia during PET experiments on small animals cannot be dispensed with. The anesthetic isoflurane offers several advantages (low metabolization rate, rapid flooding in and out, easily controllable) and is therefore frequently used. Another aspect that may have led to differences between the PET and IHC method is the partial volume effect (Rousset et al., 1998). The Focus 120 PET scanner has a resolution of ≤1.4 mm in the center of the scanner’s field of view, i.e., a partial signal loss occurs in brain structures smaller than twice the resolution of the scanner because the affected brain structures only cover a part of the scanner’s point spread function. Also, spill-over effects can arise in brain structures due to signal contributing from adjacent tissue. In the publications of Egertová et al. (2003), Harkany et al. (2003) and Cristino et al. (2006) which are used to compare with experimental PET results, the resolution of the IHC is not explicitly mentioned, but should be in the range of micrometers and therefore play a minor or no role here. It is noticeable that we observe a drastic difference between PET and IHC results for thalamus, colliculus superior, and colliculus inferior, which were categorized as “+++” for PET and “−” for IHC. Since no signal can be detected in these regions with the IHC (Egertová et al., 2003; Harkany et al., 2003), it is assumed that no receptors are present there. In addition to the general reasons for an overestimated PET signal, we believe that spill-over effects, i.e., signal contributing from adjacent tissue, are of particular importance here due to the subcortical localization of the brain regions mentioned and the high CB1 receptor density. Since we cannot explain this phenomenon in detail and cannot correct spill-over effects here, we would like to point out that the interpretation of the results of these brain regions requires special caution. Our study shows that for future intervention studies a (randomized, controlled) study design with baseline measurements is required to eliminate the overestimation of the regions. For future case-control studies we assume that these effects are not statistically significant, even if special attention must be paid to these regions, since it can be assumed that they are the same in all groups. Despite the critical aspects regarding the PET method, it has a number of advantages over *ex vivo* methods such as the IHC: it enables the assessment of the time-dependent uptake of the ligand into tissue of the living animal, also in terms of longitudinal studies, and the assessment of the entire brain instead of brain slices.

## Limitations

The first limitation concerns the use of IHC literature data for method comparison instead of experimental data for the IHC. Therefore, no intra-individual comparisons could be made for the PET and IHC methods. However, we do not consider this limitation to be serious, as experimental IHC data would only be a further confirmation of existing knowledge (see Egertová et al., 2003; Harkany et al., 2003; Cristino et al., 2006). A second limitation is the comparison of different sample shapes. For PET, we have defined volumes (slice thickness: mm) for the analyses, and the IHC is based on the analysis of brain slices (slice thickness: μm). It would hardly be possible to precisely superimpose the IHC brain slices with individual layers of PET images. In this way, the brain regions considered always remain different. Since the method comparison between PET and IHC is based on the comparison of qualitative characteristics and a ranking scale (and not on a correlation of quantitative, continuous characteristics), we believe that the error made is negligible. A third point concerns the reproducibility of the experimental PET study and the cited studies for the IHC for which we have no information. For the IHC, this would require laboratory comparisons to check and compare their measurement quality. However, as mentioned above, the distribution has already been shown in agreement in several studies (see Egertová et al., 2003; Harkany et al., 2003; Cristino et al., 2006), thus, we assume a good reproducibility. PET test-retest measurements in rats showed that the variability between the test and retest measurements was <5% (Miederer et al., 2013) so that we assume a variability in the same order of magnitude for our mice studies. Two final remarks concern the PET and IHC methods in general. These methods are suitable to measure CB1 receptor availability and protein expression; however, these parameters do not necessarily correlate with agonist-induced recruitment of G proteins and thus with functional relevance, as reviewed in detail by Busquets-Garcia et al. (2018). Furthermore, CB1 receptors are not only located at presynaptic terminals but also at postsynaptic compartments of neurons and on astrocytes, which cannot be depicted by PET or IHC. These two aspects, concerning functional relevance and imaging possibilities, must be taken into account in the evaluation and interpretation of measurement data.

## Conclusions

For the analysis of the [^18^F]MK-9470 accumulation in the mouse brain, a semi-quantitative parameter such as %ID/ml is well suited to provide a simple acquisition and analysis protocol that allows the differentiation of individual brain regions. However, care should be taken when interpreting PET results of subcortical regions, such as the thalamus, as these regions are associated with an overestimation of the PET signal. Compared to the *ex vivo* method IHC, PET makes it possible to assess the time-course of the ligand into tissue and to investigate the entire brain instead of brain slices. This preclinical [^18^F]MK-9470 study has demonstrated the radioligand’s applicability for imaging the CB1 receptor availability in the healthy mouse brain and thus offers the potential to study the endocannabinoid system in pathological conditions in mice.

## Data Availability Statement

The raw data supporting the conclusions of this article will be made available by the authors, without undue reservation.

## Ethics Statement

The animal study was reviewed and approved by Landesuntersuchungsamt Rheinland-Pfalz.

## Author Contributions

Substantial contributions to the conception and design of the work were made by IM and MS. Acquisition, analysis, or interpretation of data for the work were carried out by IM, VW, NB, PL, SM, MH and BL. All authors contributed to the article and approved the submitted version.

## Conflict of Interest

The authors declare that the research was conducted in the absence of any commercial or financial relationships that could be construed as a potential conflict of interest.
